# Increased Medical Visits and Mortality among Adults with Cardiovascular Diseases in Severely Affected Areas after Typhoon Morakot

**DOI:** 10.3390/ijerph17186531

**Published:** 2020-09-08

**Authors:** Hsin-I Shih, Tzu-Yuan Chao, Yi-Ting Huang, Yi-Fang Tu, Tzu-Ching Sung, Jung-Der Wang, Chia-Ming Chang

**Affiliations:** 1 Department of Emergency Medicine, National Cheng Kung University Hospital, College of Medicine, National Cheng Kung University, Tainan 70403, Taiwan; hshihnckutw@gmail.com; 2 School of Medicine, College of Medicine, National Cheng Kung University, Tainan 70101, Taiwan; alage@gs.ncku.edu.tw (Y.-T.H.); nckutu@gmail.com (Y.-F.T.); 3 Department of Public Health, College of Medicine, National Cheng Kung University, Tainan 70101, Taiwan; jdwang121@gmail.com; 4 Department of Urban Planning, National Cheng Kung University, Tainan 70101, Taiwan; tychao@mail.ncku.edu.tw; 5 Department of Paediatrics, National Cheng Kung University Hospital, College of Medicine, National Cheng Kung University, Tainan 70403, Taiwan; 6 School of Medicine for International Students, I-Shou University, Kaohsiung 82445, Taiwan; vivian1223@isu.edu.tw; 7 Division of Geriatrics & Gerontology, Department of Internal Medicine, National Cheng Kung University Hospital, Tainan 70403, Taiwan

**Keywords:** disaster, typhoon, flood, elderly, cardiovascular diseases, cerebrovascular diseases

## Abstract

Natural disasters have negative health impacts on chronic diseases in affected populations. Severely affected areas are usually rural areas with limited basic infrastructure and a population have that has limited access to optimal healthcare after a disaster. Patients with cardiovascular diseases are required to maintain quality care, especially after disasters. A population-based case-control study enrolled adults from the National Health Insurance Registry who had ischemic heart disease and cerebrovascular disease histories and lived in the area affected by Typhoon Morakot in 2009. Monthly medical visits for acute cerebrovascular and ischemic heart diseases markedly increased at approximately 1–2 months after the typhoon. Survival analysis during the two years following the typhoon indicated a significant increase in mortality in adults with an acute ischemic heart disease history who lived in the severely affected area. Mortality hazard analysis showed that among affected adults with previous cerebrovascular diseases and acute ischemic heart diseases, patients with diabetes (adjusted hazard ratio [HR]: 1.3–1.7), Chronic Kidney Disease (CKD) (adjusted HR: 2.0–2.7), chronic obstructive pulmonary diseases (COPD) and asthma (adjusted HR: 1.7–2.1), liver cirrhosis (adjusted HR: 2.3–3.3) and neoplasms (adjusted HR: 1.1–2.1) had significantly increased mortality rates. Consequently, high-quality and accessible primary healthcare plans should be made available to maintain and support affected populations after disasters.

## 1. Introduction

The number of reported weather-related natural disasters has been increasing since the 1960s. These disasters result in over 60,000 deaths each year worldwide [1,2]. Increasingly variable rainfall patterns have increased the frequency and intensity of flooding, which has had great impacts on human health. People may be forced to move, which heightens the risk of health effects on mental disorders, communicable diseases, and chronic comorbidities. Heavy floods cause substantial infrastructure damage and lead to massive economic and personal losses for communities [3,4]. These disasters require large-scale, multinational coordination to provide urgent humanitarian aid and continuous relief [5] to the affected areas.

Severely affected areas are usually rural and coastal areas with limited basic infrastructure and a small population. Rural and coastal areas are more likely than urban areas to have an inequitably high burden due to healthcare disparities. These disparities contribute to the inadequate provision of basic healthcare services that arise from few medical facilities, a minimal number of providers, few specialty practices, and a lack of accompanying technical innovations and health promotion programs [6,7,8]. In medically underserved rural settings, people experiencing barriers to primary healthcare tend to have a low income, low education level and high rate of unemployment [9]. Patients with chronic comorbidities need to maintain a delicate balance of care to decrease complications during and after disasters. A previous study found that 24.3% of visits to emergency treatment facilities after Hurricane Katrina were for chronic diseases or related conditions [10]. Following the March 2011 earthquake and tsunami in Japan, increased mortality was observed in some patients with chronic comorbidities [11]. Increased numbers of visits for acute ischemic heart disease and stroke following natural disasters have been identified after natural disasters. Survivors also have to cope with stressors such as searching for food and shelter, relocating, crowding, financial hardship, and navigating social services [12]. Stressors from hurricanes and other natural disasters can cause chronic and acute mental stress, which can trigger cardiovascular events. Affected communities, especially those who are severely affected and in a rural area, are faced with a myriad of disparities, each posing a barrier to timely response and complete recovery from a disaster.

The Asia-Pacific region has been recognized as the region with the highest risk of major disasters [13]. Moreover, Taiwan may be the location most vulnerable to natural hazards, with 73% of its land and population exposed to three or more hazards [14]. Typhoon Morakot affected the West Pacific Region from August 6 to 11 August 2009. On August 7, it caused copious amounts of rainfall in Taiwan, peaking at 2777 mm (109.3 in). This extreme amount of rain triggered massive mudflows and severe flooding throughout southern Taiwan, causing 702 deaths and roughly US$6.76 billion in financial losses [15,16]. Almost all southern Taiwan experienced record-breaking heavy rain, and the associated flood damage required people to relocate to temporary shelters for approximately six months to one year. After Typhoon Morakot, multidisciplinary, large-scale reconstruction and relief actions were initiated. Residents who lived in the affected areas before the disaster were identified by the Ministry of Health and Welfare and received partial medical expense reimbursement for 3 months. Medical teams and clinics were established in the temporary shelters to provide primary basic healthcare in the severely affected areas for at least 6 months. A series of recovery and relief measures were implemented and initiated in the following years. To evaluate the parameters affecting health outcomes of cardiovascular and cerebrovascular diseases among different levels of affected areas (Appendix A Table A1), we conducted a longitudinal follow-up study to assess health parameters among adult residents in different areas affected by Typhoon Morakot, a major disaster.

## 2. Materials and Methods

### 2.1. Data Source

Data from the Taiwan National Health Insurance (NHI) Database from the National Health Research Institute (NHRI) was analyzed. All claims submitted by physicians to the National Health Insurance Registry must include a diagnostic code based on the International Classification of Diseases, Ninth Revision, Clinical Modification (ICD-9-CM) developed by the World Health Organization which allows the NHI Registry to verify claims and generate statistics about causes of illness and death [17]. The NHRI recorded data included residents’ demographic data, medications, treatments (including operations), and disease diagnoses at four different levels of healthcare facilities. After Typhoon Morakot, the NHI Bureau identified residents who had lived in the affected areas before the disaster and provided partial reimbursement of their medical expenses based on the Regulation “National Health Insurance Reimbursement for Medical Expenses after Typhoon Morakot”. Adult residents living in the affected area were enrolled for further analysis.

### 2.2. Study Design

A population-based longitudinal case-control study was conducted. In total, historical data of 715,244 adults who had lived in the affected areas were obtained for this study. Adult patients with healthcare facility visit records were analyzed. The index date was 8 August 2009, the date that Typhoon Morakot struck Taiwan. The adults were separated into non-elderly and elderly groups (18–64 years and 65 years and older, respectively). Patients with chronic medical conditions requiring long-term follow-up, such as diabetes mellitus (DM), hypertension, chronic kidney disease (CKD), heart disease, hyperlipidemia, chronic obstructive pulmonary disease (COPD), liver cirrhosis and neoplasms, were identified from long-term medical records, and records of their most recent follow-up visit for their chronic medical conditions were reviewed to assess their comorbidities after the disaster. The observation period of the study was from January 2008 to December 2011.

Patients with underlying medical comorbidities, including hypertension (ICD-9-CM: 401–405), DM (ICD-9-CM: 250), asthma (ICD-9-CM: 493), chronic heart failure (ICD-9-CM: 428, 410–414), COPD (ICD-9-CM: 491–496, excluding 493, 495), liver cirrhosis (ICD-9-CM: 571.5), neoplasms (ICD-9-CM: 140–239) and CKD (ICD-9-CM: 585, 582, 583.9), that were recorded before the index date were selected and noted. All medical service utilization records after the index date were reviewed. The calculated Charlson Comorbidity Index (CCI) values indicated the severity of these patients’ comorbidities. The patients’ socioeconomic statuses were based on their income records, as reported to the NHI bureau.

The rates of acute ischemic heart diseases and cerebrovascular diseases in adults who lived in the affected areas were analyzed. The study analyzed patients with acute ischemic heart disease (ICD-9-CM: 410–414), intracranial hemorrhage (ICH) (ICD-9-CM: 430–432) and ischemic stroke (ICD-9-CM: 433–437) before and after Typhoon Morakot. The above diseases that occurred after the index date were evaluated to determine patients’ health outcomes before and after Typhoon Morakot. The monthly visits of acute illnesses were measured from the patients’ medical records. For each person, the occurrence of the same event within the same one-month period was counted only once. Hospitalization records after Typhoon Morakot were also analyzed to evaluate the health hazards, heart failure (ICD-9-CM: 428); common infections such as lower respiratory tract infection (ICD-9-CM: 480–488), urinary tract infection (ICD-9-CM: 590, 595, 597, 599), and skin and soft tissue infections (ICD-9-CM: 680–686); and trauma and injury (ICD-9-CM: 850–959).

The validated definition of mortality was adapted from the NHI database and was based on the insurance status among those affected adults enrolled in our study. Mortality cases were defined as enrolled adults withdrawn due to death or critical discharge with the diagnosis of one of the most common twenty causes of death (ICD-9-CM) of National Statistics in Taiwan, and without any medical records after withdrawal date, missing for more than six months, and were disqualified as an insurance applicant of the NHI program such as immigration and the expiration of the duration of stay of aliens [18]. All causes of death for the affected adults enrolled in this study between August 2009 and December 2011 were enrolled for further survival and hazard ratio analysis.

### 2.3. Spatial-Temporal Analysis

The classification of township and metropolitan areas in Taiwan was adapted from the 2005 Taiwan Social Change Survey, which tracks and provides insights into long-term societal trends and developments in each town or metropolitan area in six prospective areas through nationally representative survey data obtained through cluster sampling; data obtained included the proportion of the working population employed in the service sector, the proportion of the industrial population, the proportion of the population 15–64 years old, the proportion of the elderly population, the proportion of the population that attained at least a bachelor’s degree, and the population density of the area. In total, three different town or metropolitan areas (urban, suburban, and rural) were included [19].

### 2.4. Covariates

In addition to age and sex, area of residence was incorporated into the study design as a demographic variable. Comorbidities considered in this study included hypertension, DM, hypertension, COPD and asthma, liver cirrhosis, and neoplasms, which were defined by major diagnosis codes with at least three months’ long-term prescriptions before the index date with relevant ICD-9-CM codes mentioned above.

### 2.5. Statistical Analysis

The distribution of the study population, which was based on demographic and disease history data such as sex, age, diabetes, chronic kidney, pulmonary, and liver diseases, living place, social economic status, cardiovascular, cerebrovascular diseases, and mortality was analyzed. Besides A case control study to evaluate time-to-event outcomes was applied. To evaluate differences among different study groups, the chi-square test was used for categorical variables, and the Student’s *t* test was used for continuous variables. The control group was matched for age and sex using propensity scoring methods. Major underlying diseases and living locations were selected from the univariate and multi-variate Cox proportional hazard regression models with backward eliminations to estimate hazard ratios of mortality among affected adults with a history of cardiovascular and cerebrovascular events. All tests of significance were 2-tailed, and a *p* value of 0.05 or less was considered statistically significant. Robust Cox models using robust sandwich variance estimators were applied considering clustering within matched sets. Moreover, stratified Cox models using stratification of the propensity scores were also adopted to treat the matched sets. The former approach resulted in an unbiased estimation of marginal hazard ratios that were compared with a biased estimation of marginal hazard ratios resulting from the latter approach. Each comparison value and its 95% confidence interval (95% CI) were also analyzed. All data management and statistical analyses were performed with SAS 9.4 software (SAS Institute, Cary, NC, USA). All statistical tests were 2-sided, and *p* values less than 0.05 were considered statistically significant.

### 2.6. Ethical Issues

All provisions of the study were performed in accordance with the principles of the Declaration of Helsinki and the Declaration of Taipei [20]. Patients’ personal information was encrypted to protect their privacy, and the electronic databases were decoded for research; therefore, the requirement for informed consent was waived by the institutional review board (IRB). This study was approved by the IRB of the study hospital (IRB No: A-ER-103-176).

## 3. Results

**Figure A1:**
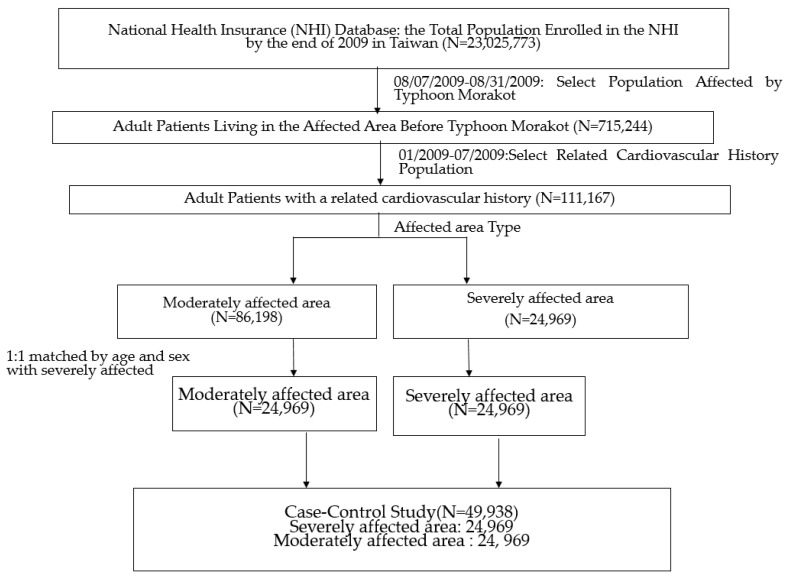
Flow diagram for patients enrolled in this study.

A total of 715,244 adult patient files were identified; 199,991 patients (28%) were in the elderly group. Female patients were predominant (398,819, 56%). The study diagram is shown in the Appendix A Figure A1. The demographic characteristics of the study population between different severity of affected areas are presented in Table 1. Compared to the adults living in the moderately affected area, those in the severely affected area before Typhoon Morakot were older, lived more in rural areas, had a higher rate of multiple underlying diseases (CCI ≥ 1: 45% vs. 41%) and cardiovascular disease history (18% vs. 15%). Besides, the adults living in the severely affected area also had higher rates of underlying chronic diseases, such as DM (16% vs. 14%), hypertension (33% vs. 26%), heart disease (12% vs. 10%), COPD and asthma (6% vs. 4%).

**Figure 1:**
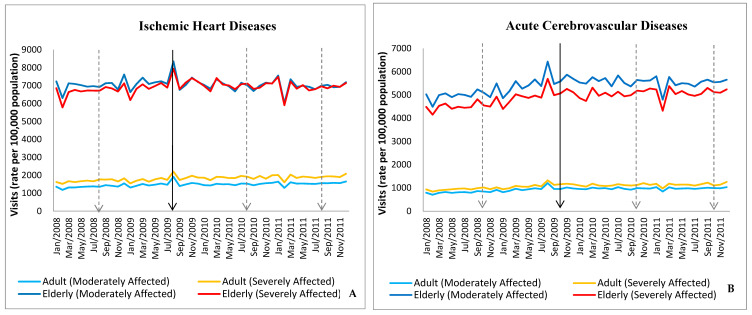
Monthly visits for acute cardiovascular and cerebrovascular events by affected adults before and after Typhoon Morakot. (**A**): Ischemic Heart Diseases; (**B**): Acute Cerebrovascular Diseases. Vertical solid lines: Index Month (August/2009); vertical dotted lines: August/2008, August/2010 and August/2011.

Figure 1 shows the number of monthly visits for acute ischemic heart disease and cerebrovascular events by affected adults before and after Typhoon Morakot, i.e., from 2008 to 2010. There was a markedly increased peak in visits in the same month Typhoon Morakot occurred in both the severely and moderately affected areas and in both elderly and non-elderly adults. Compared to that for acute ischemic heart disease, the difference in the number of monthly visits for acute cerebrovascular diseases was more prominent in the moderately affected areas, particularly in the elderly group.

The demographic data before and after matching are summarized in Table 2. After matching for age and sex, the proportions of adults living in the severely affected area with a history of cerebrovascular diseases (44% vs. 42%, *p* < 0.001), hypertension (62% vs. 58%, *p* < 0.0001), hypertension (62% vs. 58%, *p* < 0.0001), COPD and asthma (12% vs. 11%, *p* = 0.0001) was higher than those in the moderately affected area. Adults living in the severely affected area had a higher proportion of living in rural area (71% vs. 32%, *p* < 0.0001) and low-income status than those living in the moderately affected area (monthly income < 750 USD: 73% vs. 60%, *p* < 0.0001). However, there was no significant difference in the CCI score between adults living in the moderately and severely affected areas.

A mortality analysis of adults with cardiovascular and cerebrovascular disease histories was performed. Robust Cox models using robust sandwich variance estimators for clustering within matched sets were used to analyze survival after Typhoon Morakot in affected adults with cardiovascular and cerebrovascular disease histories in the moderately and severely affected areas. Before Typhoon Morakot, the robust sandwich estimation for three-year survival between moderately and severely affected areas revealed a significant difference in the survival rate of affected adults with a cardiovascular events history (*p* = 0.0047) but did not reveal a significant difference in the survival rate of affected adults with a cerebrovascular event history.

Hospitalizations after Typhoon Morakot in affected adults with cardiovascular and cerebrovascular histories before Typhoon Morakot were analyzed (Table 3). Compared to the hospitalization rate in affected adults in the moderately affected area, a slightly increased hospitalization rate was noted among affected adults living in the severely affected area after Typhoon Morakot in terms of acute ischemic heart diseases, acute cerebrovascular diseases, heart failure, infection-related diseases, trauma- and injury-related diseases (*p* < 0.0001).

To determine the important risk factors for mortality among affected adults with cardiovascular and cerebrovascular disease histories and to eliminate confounding factors, a multivariate stratified Cox proportional hazards regression model was estimated (Table 4). Among affected adults with a cerebrovascular disease history, patients with diabetes (adjusted HR: 1.40, 95% CI: 1.29–1.52, *p* < 0.0001), CKD (adjusted HR: 2.05, 95% CI: 1.81–2.33), COPD and asthma (adjusted HR: 1.89, 95% CI: 1.72–2.08, *p* < 0.0001), liver cirrhosis (adjusted HR: 2.25 95% CI: 1.79–2.79, *p* < 0.0001) and neoplasms (adjusted HR: 1.51, 95% CI: 1.33–1.71, *p* < 0.0001) had significantly increased mortality rates. Additionally, among affected adults with acute ischemic heart disease histories, patients with diabetes (adjusted HR: 1.55, 95% CI: 1.43–1.68, *p* < 0.0001), CKD (adjusted HR: 2.40, 95% CI: 2.15–2.69), COPD and asthma (adjusted HR: 1.69, 95% CI: 1.54–1.86, *p* < 0.0001), liver cirrhosis (adjusted HR: 2.76, 95% CI: 2.29–3.32, *p* < 0.0001) and neoplasms (adjusted HR: 1.87, 95% CI: 1.67–2.09, *p* < 0.0001) had higher mortality.

## 4. Discussion

This study suggested that affected adults with acute ischemic heart diseases and acute cerebrovascular disease histories before Typhoon Morakot had an increased numbers of visits healthcare facilities for acute ischemic heart disease and acute cerebrovascular diseases two months after Typhoon Morakot in both the moderately and severely affected areas. The survival analysis suggested that affected adults with acute ischemic heart disease history before Typhoon Morakot living in the severely affected area had significantly higher mortality than those living in the moderately affected area. Compared with those of affected adults in the moderately affected area, slightly more hospitalizations were noted among affected adults living in the severely affected area after Typhoon Morakot. Increased mortality rates were observed in affected adults with cerebrovascular disease and ischemic heart disease histories who had comorbidities such as diabetes, CKD, COPD and asthma, liver cirrhosis, and neoplasms.

Increases in the numbers of hospital visits for acute ischemic heart disease and stroke following natural disasters have been reported. A medical records analysis after the Great Hanshin-Awaji Earthquake in 1995 found that acute coronary syndrome and stroke rates rapidly increased and then decreased within 7 weeks, similar to rates after the Great East Japan Earthquake in 2011 [21,22]. After Hurricane Sandy in 2012, researchers detected increases in hospitalizations and deaths due to myocardial infarction and stroke during the 2 weeks following the hurricane compared to the same 2 weeks from 5 years previously [23]. In Taiwan, the rate of hospitalization due to acute myocardial infarction increased during the 6 weeks after the Ji-Ji Earthquake in 1999, and a significantly higher number of patients were hospitalized with acute myocardial infarction during that period than during the same 6-week period in the previous year [24]. Our study revealed similar results. Increased numbers of visits for acute cardiovascular and cerebrovascular events were observed in the first two months after Typhoon Morakot. The survival analysis also indicated a significant increase in mortality in the two years following the typhoon in those with previous acute ischemic heart disease who had lived in the severely affected area. Typhoon Morakot has had significantly short- and long-term health impacts on affected adults with acute ischemic heart diseases and cerebrovascular disease histories, especially those with chronic comorbidities such as DM, CKD or end-stage renal disease (ESRD), COPD, liver cirrhosis and neoplasms.

The abrupt increase in cardiovascular diseases (CVDs) in the acute phase was likely due to psychosocial and posttraumatic stress caused by the disaster and inadequate response after the disaster, especially in the severely affected areas [25,26,27]. Early morbidity from some cardiovascular events was likely predominantly attributable to the psychological stress of the event2 and corresponding physiologic derangements; psychosocial factors such as missed medications, changed diet, poor living conditions, and the stress of disorders following a disaster have been noted as well [26,28,29]. Psychosocial stressors and lack of medication and a proper support system likely play a key role. After Hurricane Katrina, multiple reports indicated high rates of psychosocial stress and posttraumatic stress [30]. Survivors had to cope with stressors such as searching for food and shelter, relocating, crowding, financial hardship, and navigating social services. Events such as hurricanes and other natural disasters can cause chronic and acute mental stress, which can trigger cardiovascular events [31]. Traditional cardiac risk factors account for only half of the incidence of CVDs, with most of the remaining risk explained by psychosocial factors [31]. Our study revealed similar results and indicated a short-term increase in the numbers of visits for acute ischemic heart and cerebrovascular diseases by affected adults with cardiovascular and cerebrovascular disease histories after Typhoon Morakot. After matching, increased two-year mortality was observed in severely affected adults with an acute ischemic heart disease history, highlighting the importance of the care of high-risk populations with cardiovascular diseases, especially those residing in severely affected rural areas. However, our study also suggested that affected populations with more co-morbidities (i.e., liver cirrhosis, CKD, COPD, asthma and neoplasms other than cardiovascular disease) have higher all-cause mortality hazards. The increased all-cause mortality occurred when patients with multimorbidity and pre-existing cardiovascular diseases such as stroke and ischemic heart diseases had increased risk of complications from either underlying diseases or comorbidities [32,33,34,35,36,37].

Health disparities are differences in the health statuses of specific populations and the general population. Health disparities are usually defined as “a particular type of health difference that is closely linked with social, economic, and/or environmental disadvantages” [8,38]. Although medical teams and acute social and financial relief programs are provided to affected populations in affected areas, affected populations with chronic comorbidities in severely affected areas still have an increased risk of mortality. Specialty and subspecialty healthcare services are usually less likely to be available in rural areas, and rural areas are less likely to offer specialized and highly sophisticated or high-intensity care than suburban or urban areas. Reliable transportation to healthcare facilities might also be a barrier for rural residents due to long distances, poor road conditions, and the limited availability of public transportation options in rural areas. These exacerbate problems for rural patients seeking specialized care who are required to travel significant distances for treatment [39]. Following a disaster, medical infrastructure usually becomes overwhelmed with acute injury and illness patients [40,41]. If chronic diseases controlled, preexisting chronic health problems can quickly become acute in nature, and these diseases have been linked to increased mortality in vulnerable populations in the wake of a disaster [40]. Chronic disease within the context of a disaster might have a bidirectional effect, whereby initial acute disorders may advance to long-term illnesses if insufficiently treated. This effect creates a “secondary surge” in required medical treatment long after the event and amplifies health disparities among medically underserved populations. The secondary surge of chronic diseases after a disaster coupled with inherent healthcare disparities, such as those commonly found in medically deprived rural areas, makes access to routine healthcare very difficult during the recovery phase [7]. Our data revealed similar results. Severely affected areas were more likely to comprise households with low socioeconomic levels. Patients with underlying comorbidities had higher mortality rates and morbidities after Typhoon Morakot that those without underlying comorbidities. Affected adults with an acute ischemic heart disease history before Typhoon Morakot living in severely affected areas had substantially increased hospitalization and mortality rates, highlighting the importance of medical accessibility. Most of the acute medical teams departed the severely affected areas approximately 6 months after Typhoon Morakot. Although relocation villages with permanent houses and healthcare centers were established within one year after Typhoon Morakot in the severely affected area, the accessibility of public transportation from these relocation villages to advanced cardiovascular care centers was limited. Accessibility limitations have great negative impacts on affected adults with cardiovascular risks, resulting in negative health outcomes.

The effects of spatial disparities after a disaster could be improved by the adoption of an improved universal healthcare system. Previous studies have suggested that universal healthcare systems substantially reduce socioeconomic inequalities in primary care access and quality but lead to only modest reductions in disparities in healthcare outcomes [42,43,44]. The Sendai framework for disaster risk reduction 2015–2030 aims to achieve substantial reductions in disaster risks and losses by enhancing the resilience of national health systems; corresponding efforts include strengthening the development and implementation of inclusive policies and social safety-net mechanisms and ensuring access to basic healthcare services, with the ultimate aim of eradicating poverty [45]. High-quality and accessible universal healthcare systems could maintain and support affected populations after disasters and are regarded as an important aspect of community resilience [46]. In order to be well-prepared for the foreseeable catastrophic natural events caused by the climate change, after Typhoon Morakot, a series of laws have been enacted including the most important “Spatial Planning Act 2016” [47] (state of the new climate-adaptive and environmental-oriented land use policy). In 2018, the Ministry of the Interior announced the implementation of the National Spatial Plan in accordance with the law and further emphasized on prioritizing the conservation of environmentally sensitive areas as well as important public facilities including medical services in rural and coastal areas. In addition, the United Nations Sustainable Development Goals (SDGs) [48] (United Nations, 2015) form the basis of spatial plans at all levels to ensure the health inequality and urban-rural gaps are taken into account in future land use policies. At the same time, more comprehensive affected area response plans including early evacuation of vulnerable groups and more medical and mental resources deployments before and after the heavy rainfall in susceptible areas were implemented to decrease the vulnerabilities and increase the resilience of the communities [49].

This study has several strengths. First, the large sample of population-based data covering almost the entire population affected by this disaster provided a large enough sample size for matching. Second, in addition to the use of traditional analyses, stratified Cox models and robust Cox models using robust sandwich variance estimators considering clustering within matched sets were employed. The latter approach resulted in unbiased estimations of marginal hazard ratios that could be compared with biased estimations of marginal hazard ratios resulting from the former approach.

Some limitations also need to be mentioned. The limitations of this study include the fact that the clinical data were collected from a registry; thus, completely verifying the data was impossible. Additionally, the registry does not include non-affected adults as reference group and provide detailed cardiovascular risk information regarding smoking habits, alcohol consumption, body mass index, physical activity, and family history, which are potential confounding factors in the analysis of chronic diseases and populations affected by disasters. The estimates of mortality among patients in this study were not verified with death certificates; however, the missing for more than 6 months is closely related to death and the proportion of disqualifications as an insurance applicant is negligible given the fact that foreigners only constitute around 2% of all insured individuals of Taiwan’s NHI from the insurance service database. The misclassification rate of the estimated mortality method was <2.37%, and the method we used has been adapted from some previous studies [50,51]. Sex and age were chosen to match in these two groups to minimize the effects of comorbidities in the elderly. Although the underlying diseases between the two groups were still different, the CCI between the two groups after matching was similar and revealed similar comorbidities between groups. Hypertension is known as an important risk factor for cardiovascular death during the acute phase of a disaster. In most patients, the increases in clinic blood pressure and self-measured blood pressure are transient, and the blood pressure levels return to the pre-disaster baseline levels within 4 weeks. The blood pressure levels should be monitored and the dose of antihypertensive medication should be reconsidered every 2 weeks during the disaster situation [52]. An inverse relationship between diastolic pressure and adverse cardiac ischemic events (i.e., the lower the diastolic pressure the greater the risk of coronary heart disease and adverse outcomes) has been observed in numerous studies. This effect is even more pronounced in patients with underlying coronary artery disease (CAD) [53]. Previous studies also indicated uncontrolled hypertension increased risk of all-cause and cardiovascular disease mortality but no significant differences were identified between normotensives, and treated and controlled hypertensives [54]. Non-adherence to antihypertensive medication increased the risk of all adverse health outcomes, including all-cause mortality and hospitalization for cardiovascular diseases such as myocardial infarction, heart failure and stroke [55,56]. The hypertension-mortality risk would be significant reduced if good adherence to anti-hypertension agents is achieved [57,58]. Our study suggested that hypertension did not increase all-cause mortality within two years among affected adults with an ischemic heart disease history after Typhoon Morakot; patients with hypertension have lower mortality hazard might be because patients marked as hypertension received regular anti-hypertensive agent treatment with relative good adherence and blood pressure levels. Further longer follow-up studies should be considered to observe the effects. The monthly visits and mortality rates after this disaster might be underestimated if affected adults were not enrolled in the national healthcare system or did not have medical records during the study period. Furthermore, the areas covered in the spatial-temporal analysis were based on the primary medical facilities that were most commonly used by the residents of the affected areas after the disaster. It was difficult to locate all the affected individuals because some patients visiting these medical facilities might not have been residing in the same area they did before the disaster. The database did not include the unaffected population as the reference group in evaluating the baseline status of mental health before and after Typhoon Morakot. The effects of Typhoon Morakot are difficult to evaluate precisely. Moreover, classifications of township development and the proportions of elderly individuals in the populations were based on the 2005 Taiwan Social Change Survey (Round 5), which primarily considered socioeconomic change and township development in Taiwan; thus, these results may not apply to other spatiotemporal analyses of the relationships between socioeconomic factors and diseases. However, data from the national census, such as the proportion of elderly individuals in the population and household income, were acquired to achieve optimal validity. Finally, nondifferential misclassification of diseases in the registry at baseline might lead to bias toward the null.

In conclusion, the health status of patients with histories of acute cerebrovascular and cardiovascular diseases before Typhoon Morakot was negative effected in both the short term and long term after Typhoon Morakot. The increase in mortality was predominant among the elderly population and was associated with comorbidities and living in the severely affected area. High-quality and accessible universal healthcare systems are important to maintain and support affected populations after disasters. Further long-term spatial and socioeconomic analyses of the health of affected populations with chronic diseases are warranted to build community resilience with optimal deployment, beneficial healthcare strategies and a collaborative framework.

## Figures and Tables

**Table 1:** Demographic and clinical characteristics of adults living between different severity of affected areas before Typhoon Morakot.

Characteristics	Adults Living in the Affected Area			
	Moderately Affected	Severely Affected	Total	Chi^2^-Test *p* Value
	(*n* = 574,089)	(*n* = 141,155)	(*n* = 715,244)	
	No. (%)	No. (%)	No. (%)	
**Sex**				
Female	321,697 (56)	77,122 (55)	398,819 (56)	<0.0001
**Age (years)**				
Mean ± SD *	51.69 ± 17.87	55.29 ± 17.64	52.40 ± 17.88	
**Age Group**				
Elderly	151,801 (26)	48,190 (34)	199,991 (28)	<0.0001
**Related Cardiovascular History (January 2008–July 2009)**				
Either of the following	86,198 (15)	24,969 (18)	111,167 (16)	<0.0001
Ischemic heart diseases	59,226 (10)	17,093 (12)	76,319 (11)	<0.0001
Acute cerebrovascular diseases	36,559 (6)	11,003 (8)	47,562 (7)	<0.0001
**Socioeconomic Status (USD/month)**				
Low < $750 USD	301,416 (53)	93,431 (66)	394,847 (55)	
**Location**				
Urban	150,232 (26)	0 (0)	150,232 (21)	<0.0001
Suburban	276,951 (48)	47,890 (34)	324,841 (45)	
Rural	146,906 (26)	93,265 (66)	240,171 (34)	
**CCI ^†^**				
0	338,653 (59)	77,717 (55)	416,370 (58)	<0.0001
1–2	183,175 (32)	49,034 (35)	232,209 (32)	
>2	52,261 (9)	14,404 (10)	66,665 (9)	
**Underlying diseases**				
DM	79,653 (14)	21,886 (16)	101,539 (14)	<0.0001
Hypertension	143,332 (26)	45,940 (33)	189,272 (26)	<0.0001
CKD	12,959 (2)	3058 (2)	16,017 (2)	0.04
Heart disease	57,835 (10)	17,171 (12)	75,006 (10)	<0.0001
COPD & asthma	25,612 (4)	8116 (6)	33,728 (6)	<0.0001
Liver cirrhosis	9570 (2)	2007 (1)	11,577 (1)	0.02
Neoplasms	32,304 (6)	7341 (5)	39,645 (6)	0.01

* Standard deviation; ^†^ Charlson Comorbidity Index. DM: Diabetes mellitus; CKD: Chronic kidney disease; COPD: Chronic obstructive pulmonary diseases.

**Table 2:** Demographics of affected adults before and after matching.

Characteristics	Adult Patients with a Related Cardiovascular History (January 2008–July 2009)					
	Pre-Match			Post-Match		
	Moderately Affected	Severely Affected		Moderately Affected	Severely Affected	
	(*n* = 86,198)	(*n* = 24,969)	Chi^2^ Test	(*n* = 24,969)	(*n* = 24,969)	Chi^2^ Test
	No. (%)	No. (%)	*p* Value	No. (%)	No. (%)	*p* Value
**Sex**						
Female	45,030 (52)	13,257 (53)	0.02	13,257 (53)	13,257 (53)	1.00
**Age (years)**						
Mean ± SD *	66.84 ± 12.76	67.72 ± 12.37		67.52 ± 12.63	67.72 ± 12.37	
**Age Group**						
Elderly	51,724 (60)	15,814 (63)	<0.0001	15,814 (63)	15,814 (63)	1.00
**Related Cardiovascular History (January 2008–July 2009)**						
Ischemic heart disease	59,226 (69)	17,093(68)	0.45	17,158 (69)	17,093 (68)	0.53
Acute cerebrovascular diseases	36,559 (42)	11,003 (44)	<0.0001	10,568 (42)	11,003 (44)	<0.0001
**Socioeconomic Status (USD/month)**						
Low < $750 USD	51,230 (59)	18,169 (73)	<0.0001	14,985 (60)	18,169 (73)	<0.0001
**Location**						
Urban	21,279 (25)	0 (0)		6213 (25)	0 (0)	<0.0001
Suburban	36,826 (42)	7267 (29)	<0.0001	10,650 (43)	7267 (29)	
Rural	28,093 (33)	17,702 (71)		8106 (32)	17,702 (71)	
**CCI ^†^**						
0	17,928 (21)	4918 (20)	0.0007	5102 (20)	4918 (20)	0.11
1–2	44,003 (51)	12,911 (52)		12,744 (51)	12,911 (52)	
> 2	24,267 (28)	7140 (29)		7123 (29)	7140 (29)	
**Underlying diseases**						
DM	24,863 (29)	7063 (28)	0.09	7225 (29)	7063 (28)	0.11
Hypertension	49,642 (58)	15,468 (62)	<0.0001	14,430 (58)	15,468 (62)	<0.0001
CKD	4613 (5.4)	1195 (4.8)	0.0004	1372 (5.5)	1195 (4.8)	0.0003
Heart disease	41,546 (48)	12,326 (49)	0.0012	12,111 (48)	12326 (49)	0.05
COPD & asthma	8891 (10)	2933 (12)	<0.0001	2662 (11)	2933 (12)	0.0001
Liver cirrhosis	1946 (2.3)	424 (1.6)	<0.0001	553 (2.2)	424 (1.7)	<0.0001
Neoplasms	6520 (7.6)	1748 (7.0)	0.0028	1933 (7.7)	1748 (7.0)	0.0015

* Standard deviation; ^†^ Charlson Comorbidity Index. DM: Diabetes mellitus; CKD: Chronic kidney disease; COPD: Chronic obstructive pulmonary diseases.

**Table 3:** Hospitalizations after Typhoon Morakot in affected adults with preexisting histories of cardiovascular and cerebrovascular diseases before Typhoon Morakot.

Characteristics	Adult Patients with a Related Cardiovascular History (January 2008–July 2009/)			
	Post-Match			
	Moderately Affected (*n* = 24,969) No. (%)	Severely Affected (*n* = 24,969) No. (%)	OR 95% CI	Chi^2^-Test *p* Value
Acute ischemic heart diseases	2607 (10)	2896 (12)	1.096 * (1.037–1.16)	<0.0001
Acute cerebrovascular diseases	1852 (7.4)	2118 (8.4)	1.129 * (1.058–1.205)	<0.0001
Heart failure	1399 (5.6)	1633 (6.5)	1.152 * (1.07–1.24)	<0.0001
Infection	4661 (19)	5301 (21)	1.123 * (1.075–1.173)	<0.0001
Trauma and injury	1238 (4.9)	1456 (5.8)	1.161 * (1.074–1.255)	<0.0001

* *p* < 0.05.

**Table 4:** Hazard ratio of all-cause mortality in affected adults with previous histories of cerebrovascular and ischemic heart diseases before Typhoon Morakot.

	Cerebrovascular Disease History ^†^ (*n* = 21,571)				Ischemic Heart Disease History ^†^ (*n* = 34,251)			
	Univariate Analysis		Multivariate Analysis		Univariate Analysis		Multivariate Analysis	
	HR	95% CI	aHR	95% CI	HR	95% CI	aHR	95% CI
Location (Ref = Nonrural)								
Rural	0.93	0.86–1.00			1.01	0.93–1.09		
DM (Ref = No)								
Yes	1.43 *	1.32–1.56	1.40 *	1.29–1.52	1.58 *	1.46–1.71	1.55 *	1.43–1.68
Hypertension (Ref = No)								
Yes	0.96	0.88–1.04			0.85 *	0.79–0.92	0.84 *	0.77–0.91
CKD (Ref = No)								
Yes	2.24 *	1.97–2.54	2.05 *	1.81–2.33	2.60 *	2.32–2.91	2.40 *	2.15–2.69
COPD & asthma (Ref = No)								
Yes	1.88 *	1.71–2.07	1.89 *	1.72–2.08	1.69 *	1.54–1.86	1.69 *	1.54–1.85
Liver cirrhosis (Ref = No)								
	2.35 *	1.88–2.93	2.23 *	1.79–2.79	3.18 *	2.64–3.82	2.76 *	2.29–3.32
Neoplasms (Ref = No)								
	1.61 *	1.42–1.82	1.51 *	1.33–1.71	2.07 *	1.85–2.31	1.87 *	1.67–2.09

^†^ Enrolled variables in Cox model for cerebrovascular disease history and cerebrovascular disease history: Location, DM, Hypertension, CKD, COPD and asthma, liver cirrhosis, Neoplasm. DM: Diabetes mellitus; CKD: Chronic kidney disease, COPD: Chronic obstructive pulmonary diseases. * *p* < 0.05.

## Appendix A

**Table A1:** Affected Townships of Typhoon Morakot.

County	Severely Affected Townships
Kaohsiung County *	Jiaxian, Taoyuan, Namaxia, Maolin, Neimen, Liugui, Qishan, Dashu, Shanlin, Meinong
Pingtung County *	Jiadong, Linbian, Neipu, Sandimen, Gaoshu, Taiwu, Shizi, Chunri, Wutai, Laiyi, Mudan, Donggang Majia
Chiayi County *	Dongshi, Alishan, Meishan, Zhuqi, Fanlu, Dapu
Taitung County	Taimali, Jinfeng, Dawu, Daren, Haiduan
Tainan County *	Nanhua, Danei, Yujing, Madou, Xuejia
Nantou County	Xinyi, Ren’ai, Shuili, Guoxing
Yulin County	Gukeng, Kouhu, Yuanchang

* All townships were severely affected.
